# An Innovative Test Method for Tensile Strength of Concrete by Applying the Strut-and-Tie Methodology

**DOI:** 10.3390/ma13122776

**Published:** 2020-06-18

**Authors:** Wen-Cheng Liao, Po-Shao Chen, Chung-Wen Hung, Suyash Kishor Wagh

**Affiliations:** Department of Civil Engineering, College of Engineering, National Taiwan University, No. 1, Sec. 4, Roosevelt Road, Taipei 10617, Taiwan; b03501023@ntu.edu.tw (P.-S.C.); r07521220@ntu.edu.tw (C.-W.H.); d08521030@ntu.edu.tw (S.K.W.)

**Keywords:** tensile strength, strut-and-tie, tension test, direct tension test, splitting test

## Abstract

Tensile strength is one of the important mechanical properties of concrete, but it is difficult to measure accurately due to the brittle nature of concrete in tension. The three widely used test methods for measuring the tensile strength of concrete each have their shortcomings: the direct tension test equipment is not easy to set up, particularly for alignment, and there are no standard test specifications; the tensile strengths obtained from the test method of splitting tensile strength (American Society for Testing and Materials, ASTM C496) and that of flexural strength of concrete (ASTM C78) are significantly different from the actual tensile strength owing to mechanisms of methodologies and test setup. The objective of this research is to develop a new concrete tensile strength test method that is easy to conduct and the result is close to the direct tension strength. By applying the strut-and-tie concept and modifying the experimental design of the ASTM C78, a new concrete tensile strength test method is proposed. The test results show that the concrete tensile strength obtained by this proposed method is close to the value obtained from the direct tension test for concrete with compressive strengths from 25 to 55 MPa. It shows that this innovative test method, which is precise and easy to conduct, can be an effective alternative for tensile strength of concrete.

## 1. Introduction

Tensile strength is an important mechanical property of concrete, although its value accounts only for 7–15% of its compressive strength [1,2,3]. In the design of reinforced concrete members, the tensile strength of the concrete is generally ignored. However, the tensile strength of concrete still has significance in terms of durability and serviceability. For example, the propagation and control of cracks are highly related to the tensile strength of concrete. Ignorance towards the tensile strength of concrete may lead to the problems of serviceability and durability and it makes tensile strength an important parameter of design.

Currently, there are three common methods for measuring the tensile performance of the concrete, namely, the direct tension test, the splitting tensile test, and the flexural test. Each of these testing methods produces different results for the concrete in tension. Out of these tests, the results can be obtained directly by using the direct tension test. However, there is a high possibility of the formation of a secondary bending moment because of the improper fixation and eccentric loading of specimens in the testing assembly [4,5,6]. Hence, the applicability of this test is limited. Both of splitting tensile tests and flexural tests are indirect methods of measuring the tensile strength of concrete, and owing to this they are not reflecting the actual results and overestimate the tensile strength of concrete [7]. Furthermore, the applicability of flexural tests usually deals with simulating the bending effect, and to be used in the design of concrete pavement under vehicular traffic [8]. Some of the researchers tried to measure the direct tensile strength of concrete by using pull out mechanism and the results were found in line with the literature [9]. Another attempt was made by using epoxy adhesive [10] and the experimental results were supported by the numerical analysis in ANSYS [11]. The authors found the difficulty for predicting the possible location of failure because of the intrinsic properties of the concrete microstructure. By using the concept of biaxial stress state same as that of in a strut-and-tie model, unique shape of the specimen for evaluating the tensile strength of concrete was developed [4]. This method overestimates and underestimates the results, respectively, with the Brazilian test and the flexural test, and different fracture mechanisms were observed in the specimens.

Table 1 depicts the range of tensile strength obtained by three different methods, where $f_{c}^{\prime }$ is the concrete compressive strength. A large variation can also be observed between them. Besides, it can be understood that the tensile strength obtained by splitting tensile test and the flexural test is representing the higher values of tensile strength as compared to the direct tension test.

Given the difficulty of reflecting the actual tensile strength of concrete with existing methods, this study proposes a new test method for overcoming the shortcomings of the above three methods. The above shortcomings were tackled by redesigning the flexural test and transmission of stress in the specimen and was also studied by the finite element software ABAQUS [12]. In this method, a strut-and-tie methodology is applied to obtain the relatively uniform tensile stress at the designed tie element. Due to construction of tie element under nearly uniform tensile stress, the test results are anticipated to be analogous to that of direct tension test. Moreover, the proposed method is a material level test and its main purpose is to encounter the limitation of other tests.

The test specimen consists of a standard flexural beam with an opening in the middle span. The opening was made where the tension side of the beam representing a thin slab. As this test is extended from the flexural test, the test setup resembles the flexural test setup. The mold is simple and easy casting, while the loading method is also the same as in the flexural test. 

## 2. Existing Concrete Tensile Strength Testing Method

### 2.1. Direct Tension Test

In a direct tension test, both ends of the specimen are clamped firmly, which causes local stress concentration and possible eccentricity of loading. Due to the heterogeneous behavior of the concrete, getting damage to the specimen on both sides and other unexpected locations is also possible [4,5,13,14,15]. Some kinds of direct tension test were proposed before, including the Three Jack Solution [16], Embedded Threaded Rod Method [17], Gluing and Gripping Test [18], and Triangular Loading Frame Method [19]. However, the complex structures of the mold, labor-intensive processes and difficulty in convincing the no eccentricity of loading makes the above tests hard to be used in materials laboratories [20,21]. These above factors affect the experimental results and cause large deviations. Therefore, there are no standard specifications released by American Society for Testing & Materials (ASTM) for measuring the tensile strength of concrete directly. Academicians and researchers are mostly preferring to use splitting tensile test and flexural test over direct tension test for measuring the tensile strength of concrete.

### 2.2. Splitting Tensile Test (ASTM C496)

**Figure 1:**
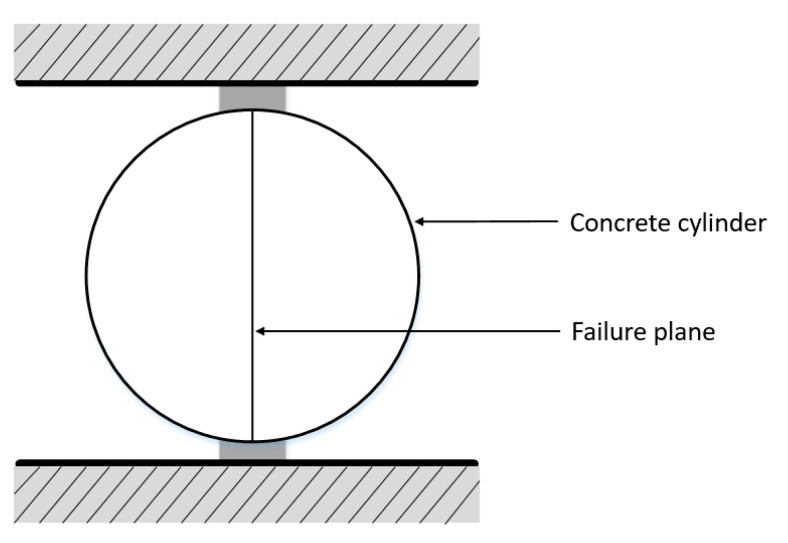
Specimen arrangement of splitting test [22].

The split tensile test is an indirect way of evaluating the tensile test of concrete. In this test, a standard cylindrical specimen is laid horizontally, and the force is applied on the cylinder radially on the surface which causes the formation of a vertical crack in the specimen along its diameter. The experimental setup for this test is shown in Figure 1. Tensile stress increases with the increase in radial compressive force and specimens deteriorate along the direction of the applied force. This test is relatively simple and needs only a standard cylindrical test specimen and a loading assembly.

**Figure 2:**
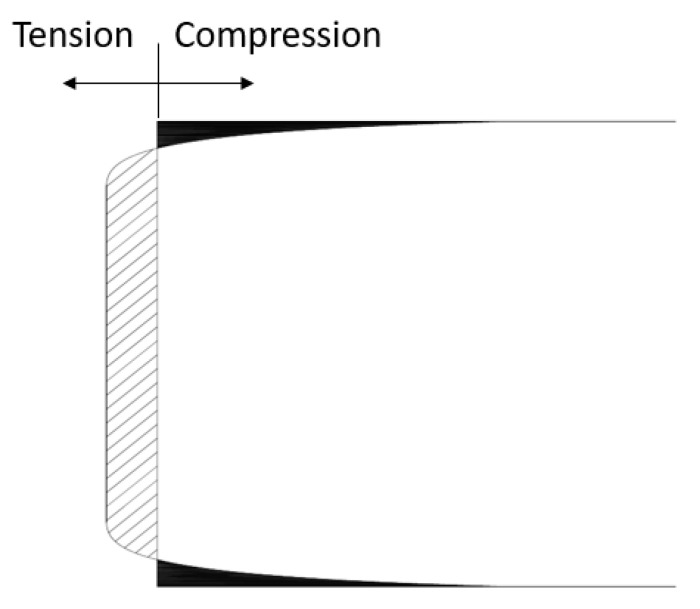
Stress distribution on vertical specimen diameter in splitting test [24].

Uneven distribution of stress under radial compressive force makes this method disadvantageous. It can be followed from Figure 2 that, the intensity of the compressive stress is greater on the top and bottom surface of the cylinder. Initially, the tensile crack appears in the central part of the cylindrical specimen and it further penetrates until the specimen reaches the maximum tensile stress. Also, as the specimen in this test is under compression therefore, the tensile test results obtained from this test are overestimating as compared with the direct tension test. Splitting tensile test overestimates the tensile strength of concrete by 10–15% [23]. Also, different maximum size of aggregates leads to the miscellaneous stress distribution [24,25,26,27,28,29]

### 2.3. Flexural Test (ASTM C78)

**Figure 3:**
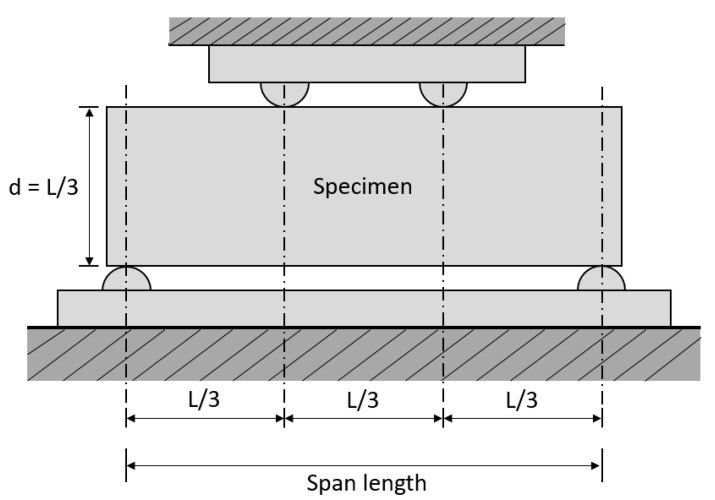
Specimen arrangement of flexural test [30].

**Figure 4:**
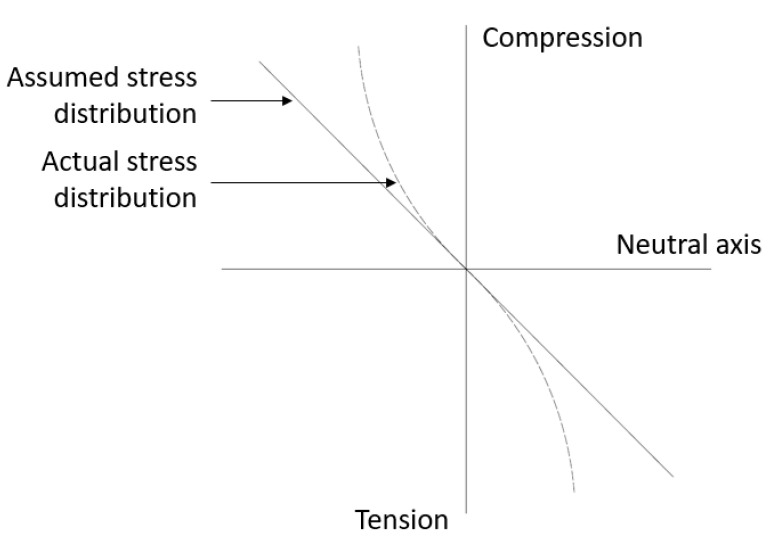
Stress distribution across specimen depth in flexural test [2].

Flexural test or modulus of rupture is another form of the indirect tension test. In this method, two loadings are applied on the beam equidistant from the center for producing the pure bending moment until the outermost fiber of the beam specimen in tension reaches the maximum tensile stress. The experimental test setup is shown in Figure 3. The upper half portion of the beam, i.e., the portion above the neutral axis, is subjected to compression while the portion below the neutral axis is subjected to tension. Also, it is assumed that there is a linear triangular variation of the stress along the section, but the actual distribution of the stresses should be parabolic instead of linear variation as shown in Figure 4. Hence, the value of the tensile strength obtained from the flexural test is higher than the actual concrete tensile test by 50–100% [2]. Moreover, in this test, the only bottommost fiber of the specimen is subjected to maximum tensile stress and it indicates that the maximum stress concentration is limited to the bottom of the specimen. Therefore, there is no uniform distribution of stresses. Nevertheless, this test is widely used because of its simple experimental test setup and easy casting of the specimens.

## 3. Experimental Program

The whole experiment includes testing and comparison of the tensile strength obtained from the aforementioned direct tension test, split tensile strength, flexural test, and a new strut-and-tie method developed in this research. The first three tests are conducted according to each testing standard. The size of the test specimen used in the strut-and-tie test is mentioned in Section 3.1 and for optimizing the width of the opening in the specimen, finite element software ABAQUS is used.

### 3.1. Design of Strut-and-Tie Beam Specimen

The new method suggested in this extraction is based on flexural test and the same loading instrument was used without any supplementary accessories. For casting the specimens, slight modifications have been made in the regular mold. 

**Figure 5:**
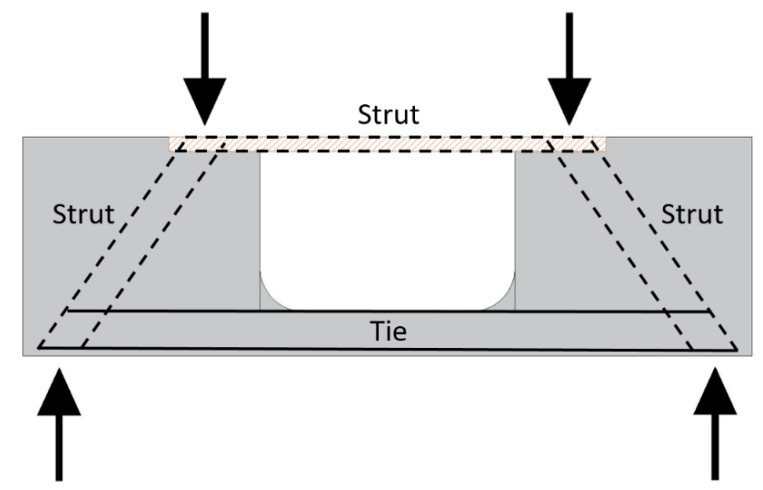
Force mechanism of strut-and-tie beam.

An opening has been provided in the middle span of the standard flexural beam specimen in order to modify the stress transmission mechanism. As shown in Figure 5, the opening has made in such a way that the bottom part of mid-span of specimen resembling a thin slab, which is representing a tie member in the test specimen; while both sides of the specimen have beam cross-section same as that in the flexural test. A groove has been made on the compression edge of the specimen for situating the steel plate. Distribution of aggregates during casting of the specimen may cause uneven distribution of stresses during testing and hence, high strength steel plate is selected on the compression side for the uniform distribution of the stresses.

The steel plate with a modulus of elasticity 200 GPa and size 300 × 150 × 10 mm was used. The minimum thickness of the concrete tie member is recommended for the more precise results. Hence, the maximum thickness of the tie member in this study is limited to 30 mm, which is 3 times the maximum size of coarse aggregate.

The behavior of the test specimen at different widths of opening and different concrete strengths was studied with a finite element modelling software ABAQUS for understanding the distribution of stresses. Through inputting compressive and tensile properties of concrete and boundary conditions in the software, specimen was divided into the number of elements and the strain and stress fields were calculated and analyzed.

**Figure 6:**
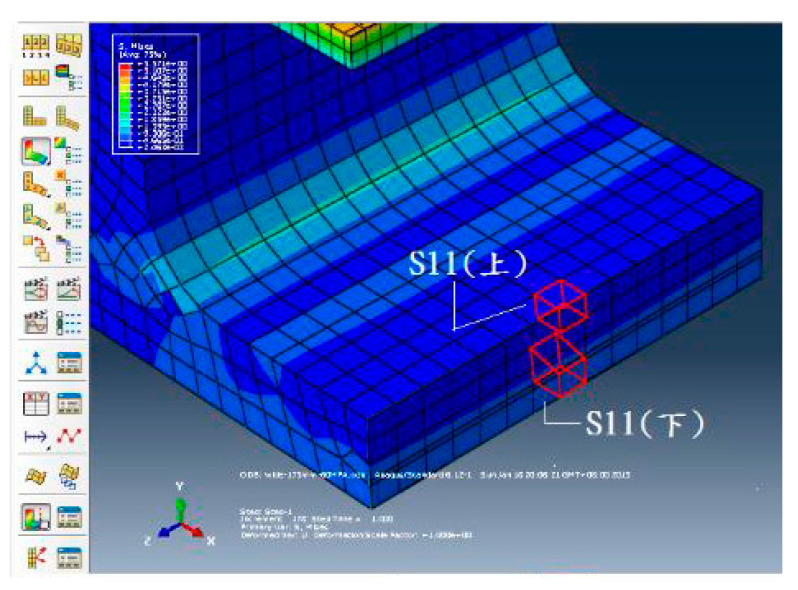
Tensile stress difference of concrete tie member in ABAQUS.

The objective of the analysis is to minimize the difference in the tensile stress between the upper and lower surfaces of the tie member so that the tie member almost reaches the evenly distributed stress condition. Figure 6 imitates the variation of tensile stresses along the cross-section of the tie member. For analysis in the ABAQUS, three different lengths of tie members were adopted and those are 150, 175, and 200 mm. The output of the analysis is displayed in Table 2. The present study does not include the influence of finite element size on the distribution of stress. The purpose of carrying out the finite element analysis is to determine the width of opening and the thickness of the tie member.

**Figure 7:**
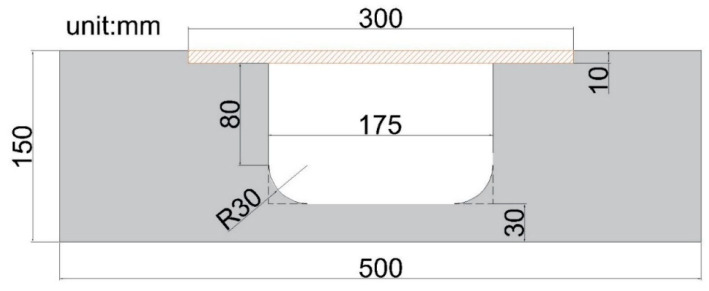
The geometry of designed strut-and-tie beam specimen.

**Figure 8:**
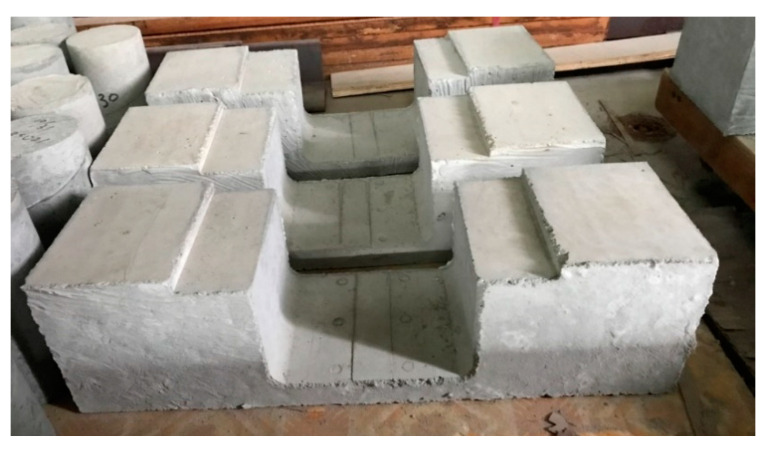
Cured strut-and-tie beam specimen.

By referring to Table 2, it can be observed that the model with a width of opening 175 mm brings about good results. This phenomenon can be explained by the fact that the difference between the distribution of the stresses on the upper and lower surfaces of the tie member are relatively small than the stresses observed in the other two widths of opening. By adopting the width of an opening of the tie member as 175 mm, the geometry of test specimen was determined. The geometry of the specimen and the casted specimens used for the testing are shown in Figure 7 and Figure 8 respectively.

### 3.2. Materials

To study the sensitivity of the test to the compressive strength of concrete, three different ordinary concrete mix proportions with design compressive strength of 30, 40, and 60 MPa were considered. Cement confirming to ASTM type 1 was used for preparing concrete mix and other ingredients include coarse aggregates with 10mm maximum aggregate size, fine aggregates, water, and superplasticizer. Detailed proportions are shown in Table 3.

### 3.3. Test Setup

**Figure 9:**
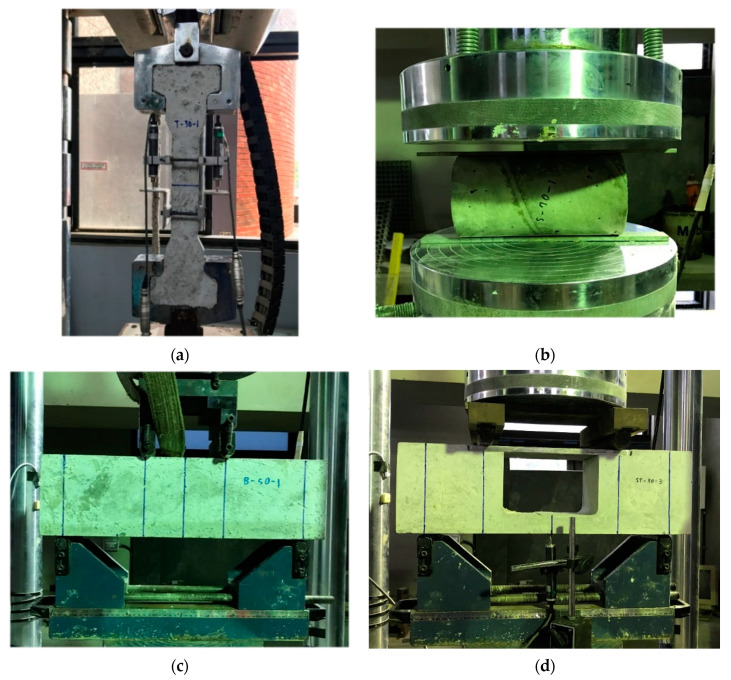
Test setup for different tests: (**a**) Direct tension test, (**b**) Splitting test, (**c**) Flexural test, and (**d**) Strut-and-tie beam test.

The casting of the specimens was done by considering at least three specimens for each of the design compressive strength for each testing method. For performing the tests, Universal Testing Machine with capacity 1000 kN was used by adapting the intensity of loading 0.01 mm/s, and specimens were loaded in the test according to the ASTM specifications. The configuration of all other tensile test setups, which are direct tension test, splitting test, flexural test and strut-and-tie beam test, are indicated in Figure 9a–d.

## 4. Calculation of Concrete Tensile Strength

### 4.1. Readily Available Tensile Strength Interpretation Formulas In a Tabular Form (Table 4)

### 4.2. Strut-and-Tie Method

**Figure 10:**
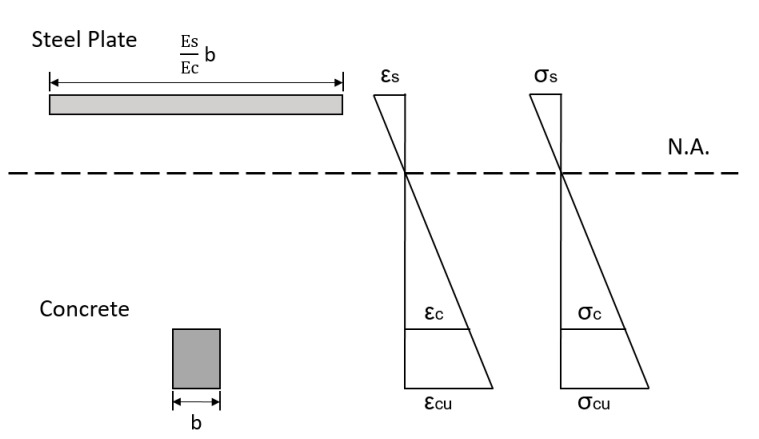
Section analysis, strain and stress distribution of strut-and-tie beam specimen.

For calculating the stresses in the bottom tie member in the strut-and-tie beam specimen, the formula from the mechanics of materials for bending was used and it is given in the Equation (4). For further calculations, same assumptions such as the beam is subjected to pure bending, the transverse section of the beam remains plane before and after bending, distribution of strain is varying linearly in the section and the material of the beam is isotropic and linearly elastic. Section of the beam includes the combination of two materials, concrete and steel, and for calculating the moment of inertia of the section, it is necessary to calculate the depth of the neutral axis first. For calculating the depth of the neutral axis, the elastic modulus of both the materials should be considered. After obtaining the values of depth of neutral axis and moment of inertia, the tensile strength for the concrete can be obtained from the following relation given in Equation (1). Figure 10 represents the stress and strain distribution in a strut-and-tie beam specimen.

(1) $$\sigma =\frac{My}{I}$$

where σ is the tensile or compressive stress, M is the moment applied to mid-span of the specimen, y is the distance from neutral axis to desired point and I is the moment of inertia.

In this strut-and-tie method, the tie member at the bottom is not uniformly under the tensile stress and it still has a little stress gradient. In agreement with Table 2, it can be revealed that for concrete with compressive strength in the range of 20–100 MPa, and with the opening of 175 mm. The difference in the tensile stress between the upper and lower surfaces of the tie member of the strut-and-tie beam specimen is less than 0.1 MPa. Hence, it can be said that the stress gradient is not evident and the distribution of stress is practically uniform.

The specimen used for this test is the same as that used in the flexural test in context with external dimensions. Hence, relatively easy production of the beams is possible in the laboratories. The following procedure describes the method for calculating the tensile strength of strut-and-tie beam.

**Figure 11:**
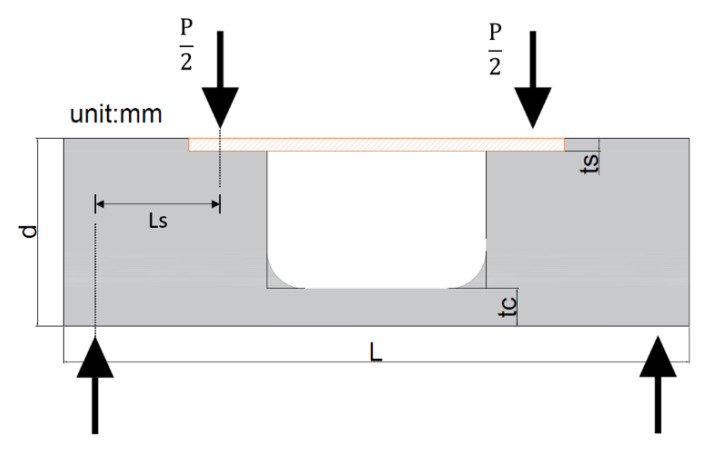
Geometry of strut-and-tie beam (width equals to b).

The geometry of the strut-and-tie specimen is shown in Figure 11. The bending moment and elastic modulus were calculated by using Equation (2) and Equation (3), respectively. Sequentially, by using Equations (4) and (5), the equivalent width of the steel plate was obtained. Later, from Equations (6) and (7), depth of neutral axis, and moment of inertia were evaluated. Finally, the tensile strength of concrete was determined by making the use of Equations (8).

(2) $$M=\frac{P}{2}\times L_{s}=\frac{L_{s}P}{2}(N-mm)$$

(3) $$E_{c}=4734\sqrt{f_{c}^{\prime }}(MPa)$$

(4) $$n=\frac{E_{s}}{E_{c}}$$

(5) $$b_{s}=n\times b$$

(6) $$\bar{y}=\frac{\sum \bar{y_{i}}A_{i}}{\sum A_{i}}=\frac{t_{s}b_{s}\times \frac{t_{s}}{2}+t_{c}b\times \left(d-\frac{t_{c}}{2}\right)}{t_{s}b_{s}+t_{c}b}(mm)$$

(7) $$I=\frac{1}{12}b_{s}t_{s}^{3}+t_{s}b_{s}\times {\left(\bar{y}-\frac{t_{s}}{2}\right)}^{2}+\frac{1}{12}bt_{c}^{3}+t_{c}b\times {\left(d-\frac{t_{c}}{2}-\bar{y}\right)}^{2}\left(mm^{4}\right)$$

(8) $$f_{ST}=\frac{M\left(d-\bar{y}\right)}{I}(MPa)$$

where $L_{s}$ is the distance between loading point and support, $E_{c}$ is the elastic modulus of concrete, $E_{s}$ the elastic modulus of steel plate, $f_{c}^{\prime }$ is the concrete compressive strength, n is the equivalent width factor, $b_{s}$ is the width of steel plate, $t_{s}$ is the thickness of steel plate, $t_{c}$ is the thickness of concrete thin plate, $\bar{y}$ is the neutral axis calculated from top fiber, and $f_{ST}$ is the tensile strength tested by strut-and-tie method.

## 5. Test Results

### 5.1. Compression Test

Table 5 gives a summary of the compression test results. Each test was performed on the concrete with three different mix proportions. From the table, it can be implied that test results on specimens with an identification number C30 performs well, while C40 and C60 are slightly lower than 40 MPa and 60 MPa.

### 5.2. Direct Tension Test

**Figure 12:**
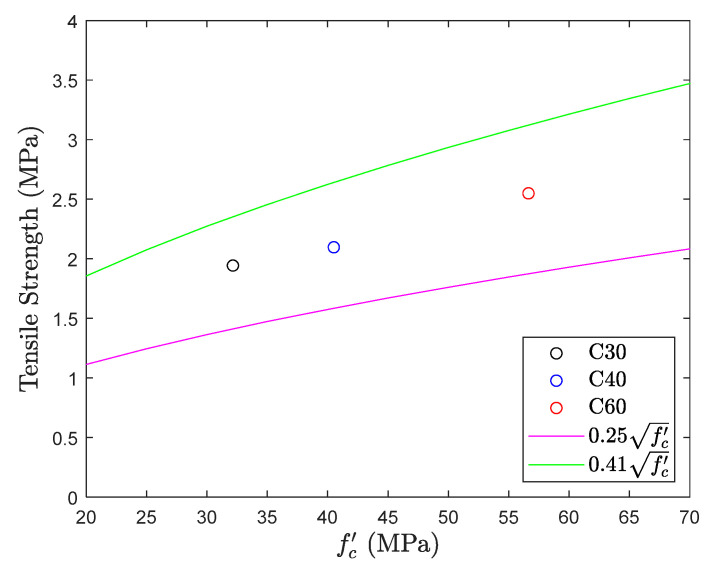
Tensile strength obtained from direct tension test.

**Figure 13:**
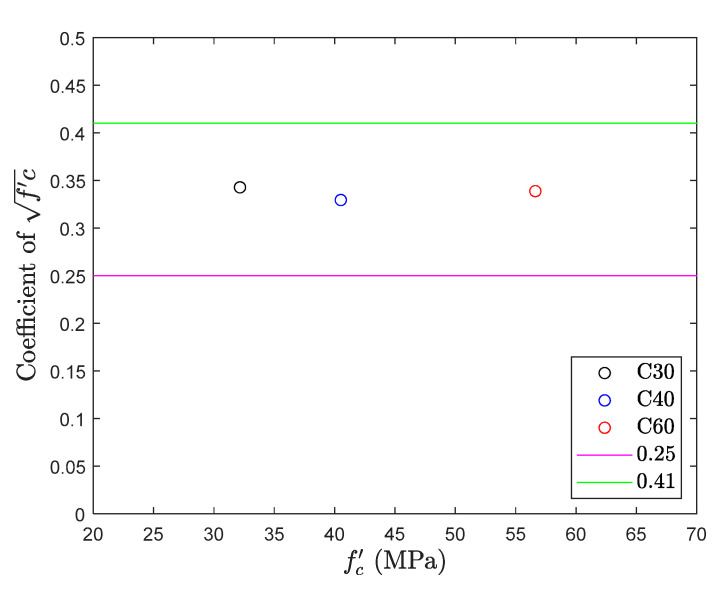
$f_{c}^{\prime }$ coefficient of direct tension test.

The results of the direct tension test are shown in Figure 12 and Figure 13. The figure shows that the tensile strength of different concrete mix proportions falls within the common range $\left(0.25\sqrt{f_{c}^{\prime }}\sim 0.41\sqrt{f_{c}^{\prime }}\right)$ [6] of the direct tension test, with an average of $0.34\sqrt{f_{c}^{\prime }}$. Although its average performance is good, it is worth noting that there is a large deviation in the experimental results. Considering proportion C40, the average tensile strength is $0.33\sqrt{f_{c}^{\prime }}$, but the difference between the maximum and minimum strength values of the specimen is $0.26\sqrt{f_{c}^{\prime }}$, which reaches up to 80% of the average strength. The same phenomenon was observed in proportion C30, and the difference was also 50% of the average strength. This shows again that there is a lot of uncertainty in the direct tension test.

The performance of the concrete under tensile force depends on the bond strength between the aggregate and mortar. The interface between the aggregate and mortar is more important in high strength concrete which may get influenced easily by the improper mixing and experimental process. Distribution of coarse aggregates may affect the test results greatly [4,26]. Therefore, the direct tension test is not widely used.

**Figure 14:**
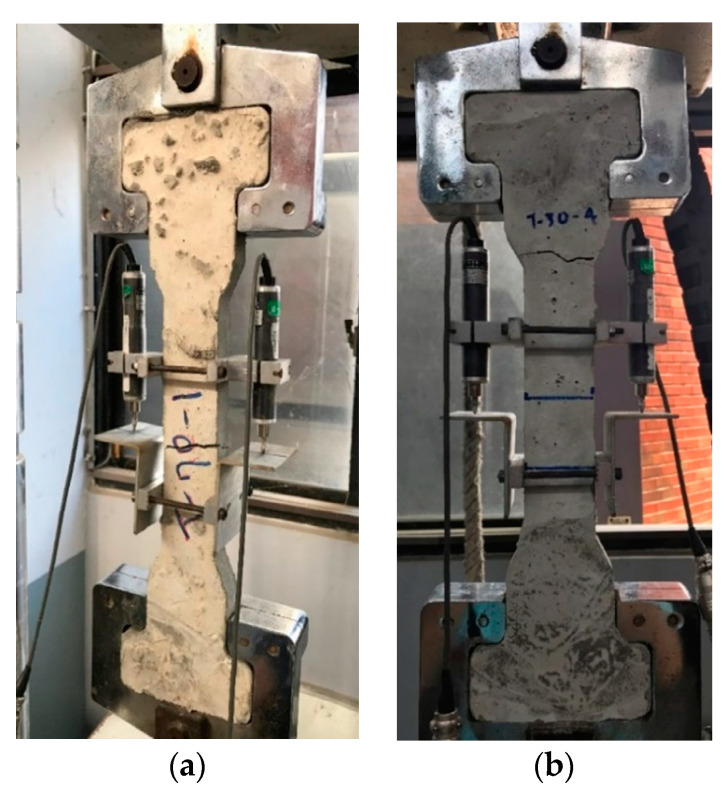
Failure mode of direct tension test. (**a**) Typical failure and (**b**) unexpected failure.

Figure 14a shows the typical pattern of failure in a direct tension test. The surface of failure is located in the central part of the specimen. This part is referred to as a critical section with the smallest cross-sectional area. As explained above, however, the tensile strength of the concrete is largely affected by the adhesive forces between cement slurry and aggregates. Hence, failure of the specimen may occur at the unexpected location as shown in Figure 14b. According to Figure 14b, it can be said that the material begins to fail at the anchorage region where the area is larger for gripping. Its larger section area could also reduce the possibility that the specimen fails here. However, it is clear that this design is not able to avoid unexpected failures. Those failures may happen due to eccentricity of loading or secondary bending moments and the above factors bring large variation and decrease the reliability of this method.

### 5.3. Splitting Test

**Figure 15:**
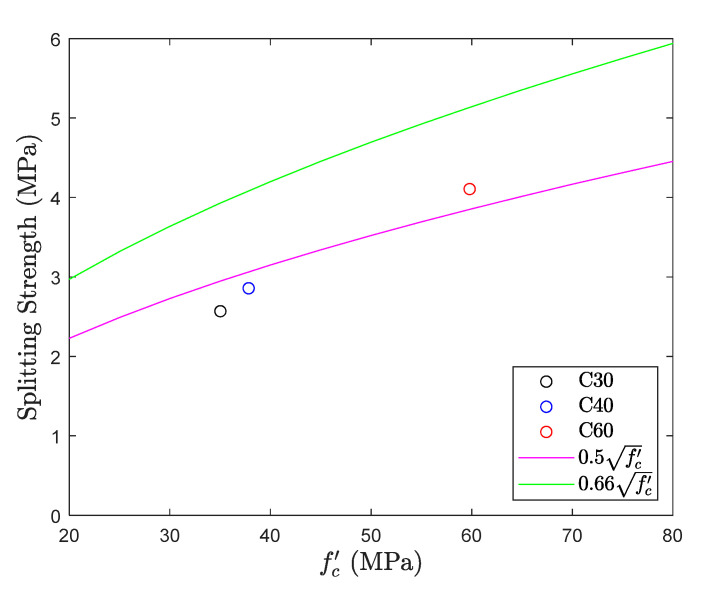
Tensile strength obtained from splitting test.

**Figure 16:**
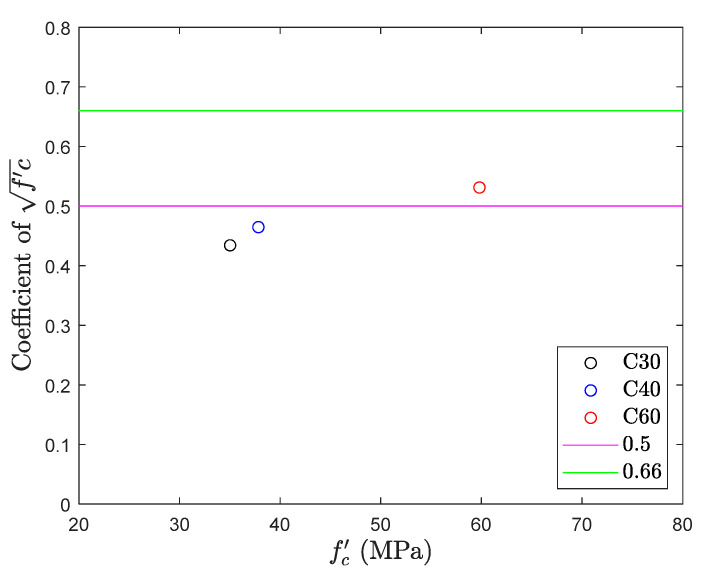
$f_{c}^{\prime }$ coefficient of splitting test.

The results of splitting tensile test are shown in Figure 15 and Figure 16. The figure also gives the top and bottom range for the tensile strength of concrete. The average splitting tensile strength of all the three different mix proportions was recorded $0.48\sqrt{f_{c}^{\prime }}$. Out of three mix proportions, C30 and C40 confer the result slightly lower than the bottom range of the tensile strength. All the results fall near the average value $0.5\sqrt{f_{c}^{\prime }}$ and no significant change was observed in the tensile strength with an increase in the design compressive strength of concrete. Also, when compared with a direct tension test, splitting tensile test with an average value $0.48\sqrt{f_{c}^{\prime }}$ is higher than the average in direct tension $0.34\sqrt{f_{c}^{\prime }}$ with a difference of 40%. A split tensile test is involved with compression and thus results cannot reveal the tensile strength of concrete.

**Figure 17:**
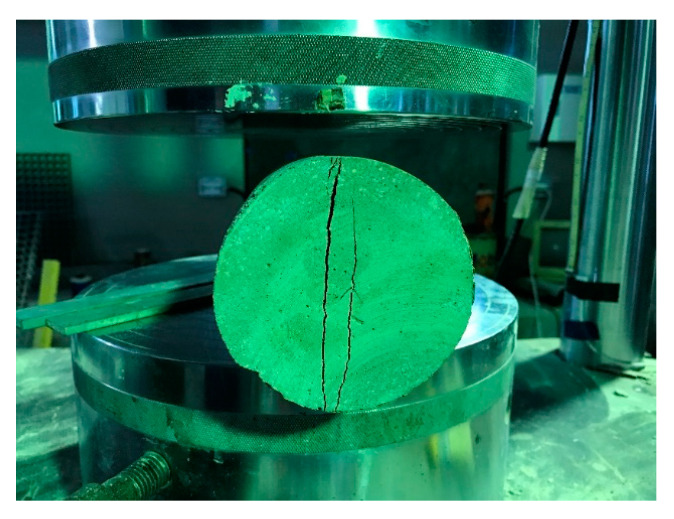
Failure mode of splitting test.

Failure pattern in the splitting tensile test is exhibited in Figure 17. The crack grows in the radial direction and finally damages the specimen by dividing it into the two parts diametrically. Sometimes, the crack may not appear along the diameter of the cylindrical specimen perfectly because of the experimental errors but the outcome of tensile test result is same as expected.

### 5.4. Flexural Test 

**Figure 18:**
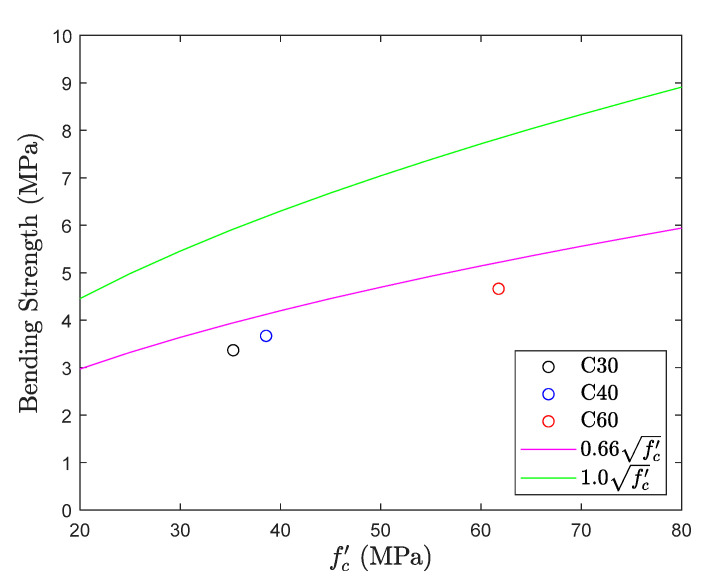
Tensile strength obtained from flexural test.

**Figure 19:**
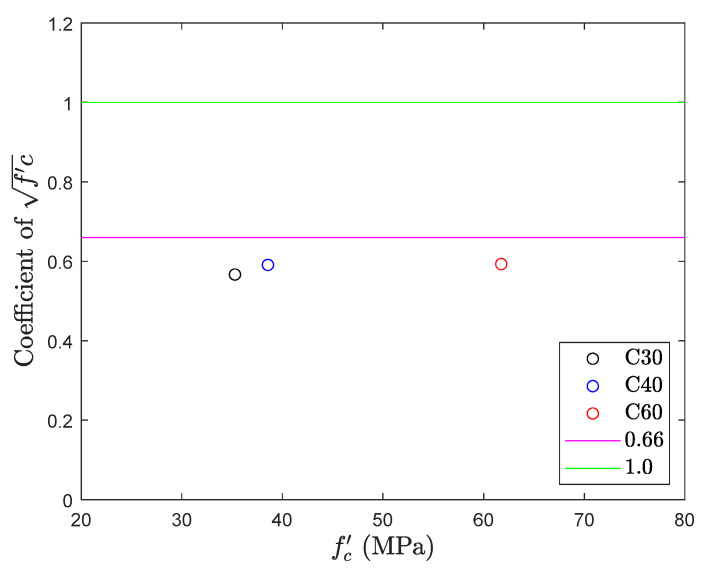
$f_{c}^{\prime }$ coefficient of flexural test.

**Figure 20:**
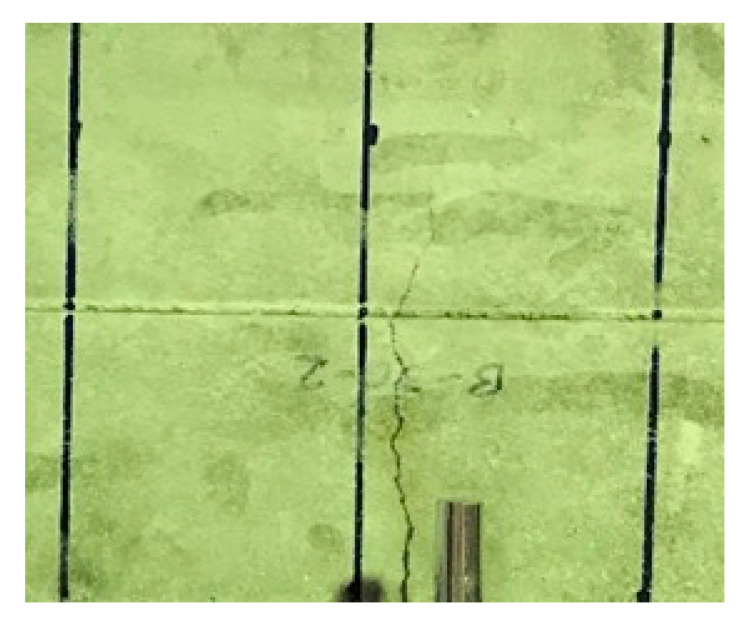
Zoom view of failure mode in flexural test.

Figure 18 and Figure 19 give the results for the flexural test. They unfold that the observations recorded for the different mix proportions of concrete in the flexural test are lower than the bottom range of the tensile strength, i.e., $0.66\sqrt{f_{c}^{\prime }}$, by indicating the average of $0.58\sqrt{f_{c}^{\prime }}$. Test results symbolize that the variability of the test results in the flexural test is relatively small while comparing with the previous test results. This is because the flexural test uses four-point loading criteria, and the test specimen has a large cross-sectional area which enables more consistent loading for developing the effective tension and compression zones. In contrast, the flexural test is still a type of compression test. Results obtained by the flexural test ($0.58\sqrt{f_{c}^{\prime }})$ are significantly higher than the direct tension test results ($0.34\sqrt{f_{c}^{\prime }})$, in terms of percentage this difference is as high as 70%. Result obtained from this test is called modulus of rupture and is different in meaning from the tensile test. Besides, the results needed to be converted from the modulus of rupture to tensile strength. Figure 20 shows the failure of the specimen during the flexural test. A crack was appeared on the central span of the tension surface and then propagated toward the top with an increase of loading and these results are in line with the Welch [31].

### 5.5. Strut-and-Tie Method

**Figure 21:**
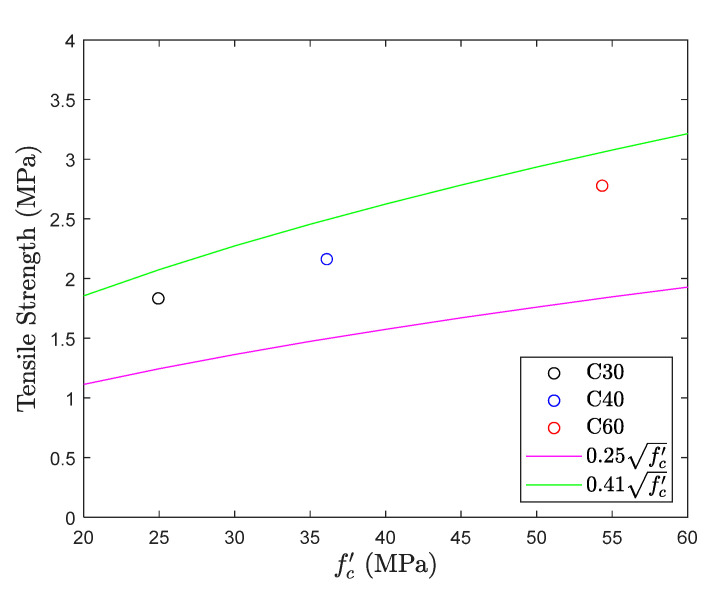
Tensile strength obtained from strut-and-tie beam test.

**Figure 22:**
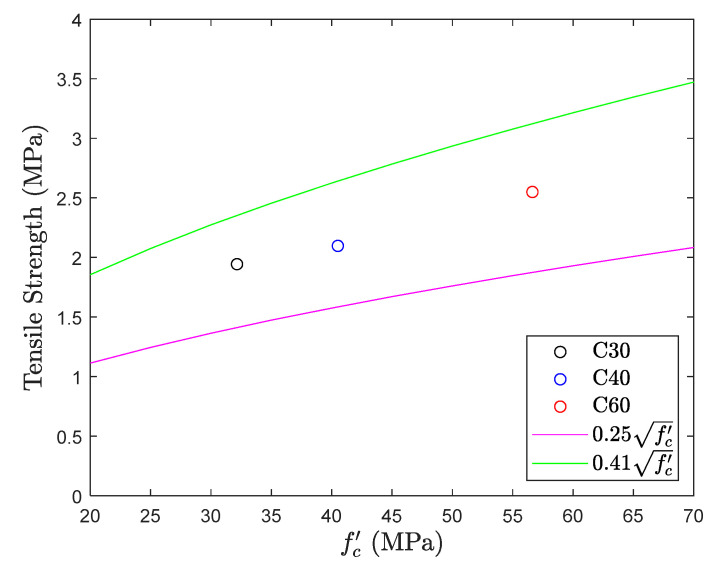
$f_{c}^{\prime }$ coefficient of strut-and-tie beam test.

This section depicts the results of the strut-and-tie method developed in this study. From Figure 21 and Figure 22, it can be observed that the tensile strengths for all the design compressive strength of concrete considered in this research are within the top and bottom range of concrete tensile strength, with an average tensile strength of $0.37\sqrt{f_{c}^{\prime }}$. The variation in test results of C30 and C60 test is only about $0.1\sqrt{f_{c}^{\prime }}\;$ which is much smaller than the difference observed in the direct tension test.

The measured tensile strength with a strut-and-tie method $\left(0.37\sqrt{f_{c}^{\prime }}\right)$ is quite close to the value obtained by using direct tension test $\left(0.34\sqrt{f_{c}^{\prime }}\right)$ with an only difference of 9%. Hence, it can be said that this method measures the concrete tensile strength with the same accuracy of the direct tension test but with fewer variations.

**Figure 23:**
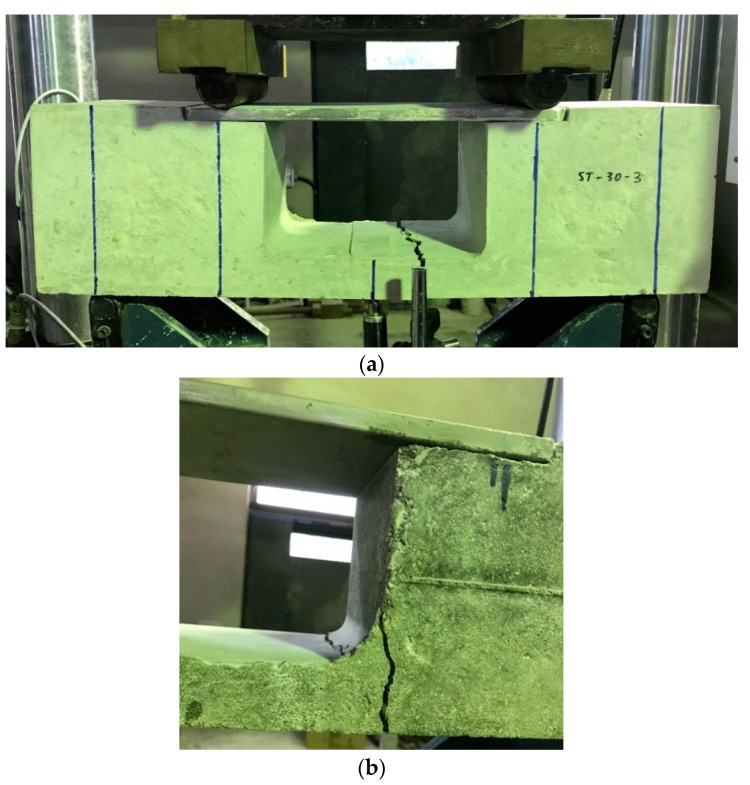
Failure mode of strut-and-tie method: (**a**) typical failure and (**b**) unexpected failure.

In Figure 22, the coefficient is relatively stable with the change of design compressive strength of concrete. In this method, deformation in the middle span of the beam is due to the bending moment and not because of the shear force, this phenomenon is also observed in the flexural test, and hence it is expected to initiate the formation of the cracks in the middle span of the test specimen. Typical failure mode in the strut-and-tie beam is shown in Figure 23a and the expected critical section of failure is located in tie member with a smaller cross-sectional area. However, because the shape of tensile member changes highly from a square to a thin slab, tensile stresses develop instantaneously, and this may make specimen fail in an unexpected way, as shown in Figure 23b. Figure 23b also reveals that the location of the failure is still in the tie member but it is close to the interface between the beam cross-section and the tie member.

The small curvature was provided at the intersection of the beam section and tie member to avoid the concentration of stresses. Nevertheless, as concrete is an anisotropic material and the distribution of aggregates is not always uniform, the presence of smooth and annular coarse aggregates may affect the bond strength between aggregate and cement paste [24,28]. The fact is that presence of maximum size of aggregate and its location may influence the test results. About 20% samples were failed near the interfacial zone. 

Table 6 summarizes the test results of four different test methods in this study and their corresponding coefficient to $\sqrt{f_{c}^{\prime }}$.

## 6. Analysis of Test Results

### 6.1. Comparison of ABAQUS Analysis and Test Result

Table 7 shows the simulation results of the computer program. The simulation was done for evaluating the tensile strength of concrete in the range of 20 to 100 MPa by using computer software. Experimental results are given in Table 5 and Table 6. The data given in Table 7 is showing the ABAQUS results for concrete with different design compressive strengths. The actual values of the concrete compressive strength (Table 5) for the strut-and-tie beam were used for interpolation with the tensile strength results from the ABAQUS to obtain the value of tensile strength. This interpolation gives a new value which represents the actual tensile strength of concrete for that particular compressive strength of concrete as shown in Table 8. From Table 8 it can be observed that the results obtained from the strut-and-tie beams are lower than the simulation results after the interpolation. However, these results are in the top and bottom range of the tensile strengths. The lower bound and upper bound values of tensile stress were obtained by following to Table 1.

This discrepancy may occur because of the inputs required in ABAQUS like the properties of the materials and accuracy of the material property ultimately affects the accuracy of the simulation. It is more difficult to obtain the stress-strain curve for the concrete in tension than concrete in compression, and the linearity and numerical stability are also poor, which makes the simulation results overestimated. In this study, only the stress-strain curve obtained from the past results of the direct tension test was used as an input in the analysis software. With the 175 mm width of the opening in the strut-and-tie beam, the difference between the stresses on the upper and lower surface of the tie member was less as shown in Table 2.

### 6.2. Comparison of Different Testing Methods

**Figure 24:**
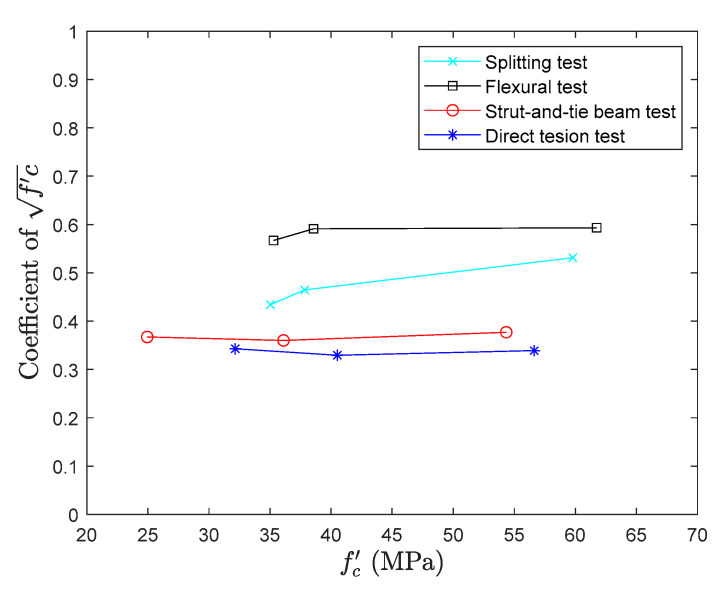
Comparisons between models of $f_{c}^{\prime }$ coefficient.

In Figure 24, the results of all the methods are presented in a graphical form. From the figure, it can be observed again that results composed by flexural test and splitting tensile test are overestimating the direct tension test results. The variation in the tensile test results is about 40% and 70%, respectively, in the splitting tensile test and flexural test and these variations are consistent with the literature. Also, in those tests, specimens are not subjected to pure tensile stress and the results needs to be converted to tensile strength through empirical formulas. Because empirical formulas are also derived from the experimental regression, there may still be errors in the conversion.

The proposed strut-and-tie method produces the results close to the direct tension test with a minimum variation. The difference between the results of the direct tension test and the strut-and-tie method is only about 9% and shows the proposed method has a good level of accuracy.

## 7. Conclusions

A comparison of the results of the different tension tests with an innovative test method by applying strut-and-tie methodology was evaluated in this article. Based on the test results and analysis of this study, the following conclusions can be drawn:

1. Three different kinds of tensile tests for concrete were studied and a new method was proposed for evaluating the performance of concrete under tension. This new method is mainly adopting strut-and-tie methodology. The performance of the concrete in tension was evaluated by using all the four methods and the results were compared to determine the feasibility of each test. The results indicated that the newly proposed test produces good test results.
2. Implementation of the strut-and-tie method is very simple and the results obtained from this method are nearly similar to the actual tensile strength of concrete. This method is advantageous in the following ways: the test does not need any special mold for casting the specimen, maintaining the test specimen is easy, loading equipment and testing setup is the same as in the flexural test.
3. The optimum width of the opening 175 mm in the strut-and-tie beam was assessed by using the software based on the Finite Element Method (ABAQUS). The thickness of the tie member, 30 mm, was decided based on the maximum size of aggregate used in the concrete. The width of the opening was adopted to ensure the fairly uniform distribution of the tensile stresses in concrete tie member on both upper and lower surfaces as in the direct tension test.
4. Tensile test results obtained by using the other three methods have their shortcomings such as direct tension test is prone to eccentricity and secondary bending moment and gives variation in results. Also, split tensile test and flexural tests both are the indirect ways of measuring the tensile strength, and specimens are not purely subjected to tension and the test results obtained from these tests overestimates the concrete tensile strength.
5. With 175 mm width of the opening, the results obtained by the strut and tie method are $\left(0.37\sqrt{f_{c}^{\prime }}\right)$ very close to the results obtained through a direct tension test $\left(0.34\sqrt{f_{c}^{\prime }}\right)$ with a minimum discrepancy of results. Experimental stability and reliability of the strut-and-tie method make it more coherent for evaluating the tensile strength of concrete.

## Figures and Tables

**Table 1:** Approximate range of concrete tensile strength obtained by different methods [6].

Testing Methods	Range of Tensile Strength (MPa)
Direct tension test	$\left(0.25\sqrt{f_{c}^{\prime }}\sim 0.41\sqrt{f_{c}^{\prime }}\right)$
Splitting tensile test	$\left(0.5\sqrt{f_{c}^{\prime }}\sim 0.66\sqrt{f_{c}^{\prime }}\right)$
Flexural test	$\left(0.66\sqrt{f_{c}^{\prime }}\sim 1.0\sqrt{f_{c}^{\prime }}\right)$

**Table 2:** The difference in tensile stress at the tie member obtained from ABAQUS analysis for different widths of opening.

Width of Opening (mm)	Design Compressive Strength of Concrete (MPa)					Analysis Result
	20	40	60	80	100	
	The difference in Tensile Stress (MPa)					
150	0.13505	0.13516	0.10999	0.10200	0.14929	
175	0.00758	0.05074	0.06396	0.03996	0.07586	Best
200	0.10306	0.20540	0.25261	0.28307	0.40291	

**Table 3:** Details of mixture proportions (kgf/m^3^).

ID	Compressive Strength Target Value (MPa)	Cement	Sand	Coarse Aggregate	Water	SP
C30	30	450	1000	700	293	2.25
C40	40	450	1000	700	225	5.85
C60	60	450	1000	700	149	9.9

**Table 4:** Tensile strength formulas [27].

Name of the Test	Formula	Description
Direct Tension Test	$f_{\mathrm{DT}}=\frac{P}{\mathrm{bt}}$	where $f_{DT}$ is the direct tension strength, P is the maximum load, b is the width and t is the thickness of the critical cross section of the specimen.
Splitting Test	$f_{\mathrm{SP}}=\frac{2P}{\pi \mathrm{ld}}$	where $f_{SP}$ is the splitting tensile strength, l is the length and d is the diameter of the cylinder specimen.
Flexural Test	$R=\frac{\mathrm{PL}}{{\mathrm{bd}}^{2}}$	where R is the modulus of rupture, L is the span length of the specimen, b is the width and d is the depth of the specimen, equals to $\frac{L}{3}$.

**Table 5:** Summary of compression test results.

	Proportion ID	Concrete Compressive Strength (MPa)
Direct tension test	C30	32.15
	C40	40.51
	C60	56.63
Splitting test	C30	35.03
	C40	37.84
	C60	59.80
Flexural test	C30	35.28
	C40	38.56
	C60	61.76
Strut-and-tie method	C30	24.94
	C40	36.10
	C60	54.34

**Table 6:** Summary of test results.

Testing Methods	ID	Test Tensile Strength (MPa)	$\mathrm{Coefficient}\;\mathrm{of}\;\sqrt{f_{c}^{\prime }}$	Average Coefficient
Direct tension test	C30	1.94	0.34	0.34
	C40	2.10	0.33	
	C60	2.55	0.34	
Splitting test	C30	2.57	0.43	0.48
	C40	2.86	0.46	
	C60	4.11	0.53	
Flexural test	C30	3.37	0.57	0.58
	C40	3.67	0.59	
	C60	4.66	0.59	
Strut-and-tie method	C30	1.83	0.37	0.37
	C40	2.16	0.36	
	C60	2.78	0.38	

**Table 7:** ABAQUS analysis result of strut-and-tie method.

	Concrete Compressive Strength (MPa)				
	20	40	60	80	100
ABAQUS analysis result (MPa)	2.05	2.84	3.51	4.06	4.55

**Table 8:** Stress comparison with different concrete compressive strength.

Testing Method	Concrete Compressive Strength (MPa)		
	24.94	36.10	54.34
	Testing Stress (MPa)		
ABAQUS analysis	2.24	2.69	3.32
Experiment result	1.83	2.16	2.78
Lower bound	1.25	1.50	1.84
Upper bound	2.05	2.46	3.02
